# Cancer

**Published:** 1885-04-20

**Authors:** 


### CANCER.

The cases of the late Senator Hill and of Gen. Grant seem to have excited increased activity in the investigation of malignant affections. We are glad to note that the articles of our correspondent, Dr. H. M. Lawson, relative to the curative power of arsenic in cancerous diseases, have attracted the attention of certain prominent observers in the Profession In this issue of our Journal may be seen an article by Dr. J. McF. Gaston, Professor of Surgery in the Southern Medical College of Atlanta, in which the efficacy of arsenic in modifying, if not in actually removing, the cancerous diathesis is forcibly illustrated. It is certainly an important point gained that a remedy is found which counteracts the destructive process of this terrible disease. It shows that the prevailing tendency on the part of the regular Profession of abandoning this class of diseases as hopelessly incurable is wrong, and gives strong ground to hope that, in arsenic, we have an agent which, heroically and properly used, may enable us to cope successfully with this awful and hitherto hopeless malady.

